# Analysis of the DC Field Emission Properties of Laser-Polished Niobium Samples

**DOI:** 10.3390/ma19153258

**Published:** 2026-08-01

**Authors:** Florian Brockner, Dirk Lützenkirchen-Hecht

**Affiliations:** Solid State Physics, Faculty 4, University of Wuppertal, Campus Grifflenberg, 42119 Wuppertal, Germany

**Keywords:** niobium, laser polishing, field emission, superconducting radio frequency

## Abstract

The field emission properties of laser-polished, fine-grained niobium sheets as the base material for an application in superconducting radio frequency cavities were investigated using a specially developed field emission scanning microscope. To investigate the influence of laser polishing on field emission properties, two surfaces were examined: an unpolished surface and a laser-polished surface, with different conditions for the field emission measurements. Scanning electron microscopy was used to characterize the surfaces before and after the field emission measurements. The field emission field strengths (onset-field $E_{E}$) were measured to quantify the electron emission over large areas. For smooth, defect-free surfaces, emission field strength values up to 1 GV/m were achieved, while untreated or defect-rich areas remained limited to around 100 MV/m or less. A local reduction of the onset field strength could be attributed to defects such as carbon contamination, pores and cracks. The field emission data obtained was categorized into four classes. This work shows that laser-polished niobium surfaces only exhibit electron field emission at field strengths above 700 MV/m, a tenfold increase over chemically or electrochemically polished samples, thus demonstrating the potential for future superconducting radio frequency cavity applications. At the same time, laser polishing needs further improvements to enable a reliable large-scale manufacturing process of superconducting radio frequency cavities.

## 1. Introduction

Modern particle accelerators such as the Large Hadron Collider (LHC) at CERN [1], the European Spallation Source (ESS) [2] and the European X-ray Free Electron Laser (XFEL) [3] are based on superconducting high-frequency resonators (SRF cavities) made of niobium. In order to achieve acceleration field gradients of 30 MV/m and more, sophisticated manufacturing and surface treatment processes have been established on both experimental and industrial levels. On a laboratory scale, field strengths of up to 50 MV/m can be achieved, which is close to the theoretical limit [4,5].

The current state of the art includes chemical polishing processes for removing surface contaminants and high-vacuum heat treatments for reducing lattice defects and residual stresses [5,6]. Processes such as buffered chemical polishing (BCP) with hydrofluoric acid and nitric acid [7], centrifugal polishing or electropolishing (EP) [8] are used to convert the technically rough surface into a smooth, mirror-like structure and to eliminate defects. Smooth, defect-free surfaces are a basic requirement for suppressing parasitic field emission, which can limit the achievable acceleration fields and reduce efficiency [9]. Finally, polishing residues must be removed, as these are an effective source of field emitters, by employing additional cleaning steps such as high-pressure water rinsing [7,8] or dry ice cleaning [10]. In recent years, laser polishing (LP) has been identified as a resource-efficient and environmentally friendly alternative to conventional chemical polishing methods [11,12]. By LP, the surface layer of the material is locally melted by a focused, high-intensity laser beam, usually under a protective gas atmosphere to avoid oxidation effects. During solidification, self-organization mechanisms in the melt result in a significant smoothing of the surface. The use of a continuous-wave (cw) laser is commonly referred to as macro polishing, as it enables the reduction of large-scale surface irregularities and long-wavelength roughness components. Conversely, micro polishing is performed using a pulsed laser, targeting the reduction of micro-scale roughness features [13]. Previous studies have focused on optimizing process parameters and fundamentally evaluating the resulting field emission properties [14].

In general, it was observed that laser-polished metal surfaces have been shown to exhibit increased field emission field strengths [14,15]. The aim of this work is to provide a detailed analysis of the local field emission characteristics of laser-polished niobium surfaces.

## 2. Theory of Field Emission

**Figure A1:**
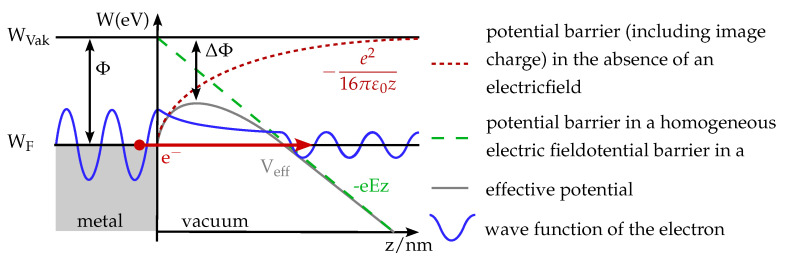
Schematic illustration of the field-induced lowering of the surface potential barrier. The effective work function Φ is reduced by the external field potential −eEx and the image-charge correction term $-e^{2}/16\pi \varepsilon_{0}x$ arising from the induced mirror charge.

Field emission refers to the emission of electrons from a surface under the influence of an applied electric field *E*. The emission process is described by quantum-mechanical tunneling of electrons through a surface potential barrier. The applied electric field lowers both the height and width of the potential barrier through the field potential −eEx and the image-charge potential $-e^{2}/\left(16\pi \varepsilon_{0}x\right)$ (cf. Figure A1), thereby enabling electron tunneling [16,17,18,19].

Surface imperfections such as micro-protrusions, particles or chemical impurities locally enhance the electric field and therefore increase the probability of electron emission [10,20]. This local field enhancement is commonly described by the field enhancement factor β, relating the macroscopic applied field *E* to the local surface field $E_{\mathrm{local}}$ according to

(1) $$\begin{array}{c} E_{\mathrm{local}}=\beta \;E. \end{array}$$

Using this relation, the field-emission current can be described by the simplified Fowler–Nordheim equation

(2) $$\begin{array}{c} I_{\mathrm{FN}}\left(E\right)=\frac{A\;S{\left(\beta \;E\right)}^{2}}{\Phi }\exp\left(-\frac{B\;\Phi^{3/2}}{\beta E}\right), \end{array}$$

where *S* denotes the effective emission area, Φ is the work function, and

(3) $$\begin{array}{c} A=\frac{e^{3}}{8\;\pi \;h},\;\;B=\frac{4\sqrt{2\;m_{e}}}{3\;\hbar \;e} \end{array}$$

are Fowler–Nordheim constants. Although this simplified expression successfully describes the emission behaviour of many field emitters, deviations may occur due to surface activation, interactions between neighbouring emitters or electrical breakdown at high electric fields [21].

Taking the natural logarithm of Equation (2) yields the linear Fowler–Nordheim representation

(4) $$\begin{array}{cc} \ln\left(\frac{I_{E}}{E^{2}}\right)\; & =a\;\frac{1}{E}+b, \end{array}$$

with

(5) $$\begin{array}{cc} a & =-\frac{B\;\Phi^{3/2}}{\beta }, \end{array}$$

(6) $$\begin{array}{cc} b & =\ln\left(\frac{A\;S\;\beta^{2}}{\Phi }\right). \end{array}$$

According to Fowler–Nordheim theory, electron emission therefore results in a linear relationship in Fowler–Nordheim coordinates [19]. The parameters *a* and *b* correspond to the slope and intercept of the linear regression, respectively. The field enhancement factor and the effective emission area are subsequently obtained from

(7) $$\begin{array}{cc} \beta & =-\frac{B\;\Phi^{3/2}}{a}, \end{array}$$

(8) $$\begin{array}{cc} S & =\frac{\Phi \;\exp(b)}{A\;\beta^{2}}. \end{array}$$

In the present work, Fowler–Nordheim analysis is employed to determine the field enhancement factor and the effective emission area of untreated and laser-polished niobium surfaces from the measured emission current as a function of the applied electric field.

Porshyn et al. [15] showed that laser micro-polishing of chemically pre-polished niobium surfaces at high fluence and high pulse numbers induces surface morphology that are emission-active at comparatively low operating fields of (70−190) MV/m, whereas smoother surfaces produced at lower fluence and reduced pulse counts support field emission at significantly higher field strengths of (650−940) MV/m. Selective measurements carried out on macro- and micro-LP surfaces showed, that the surfaces may have field strengths of more than 800 MV/m [14].

It was concluded that LP removes micro-peaks that lead to local field enhancement, thereby allowing substantially higher electric fields without any (parasitic) field emission. However, the field emission properties of macro- and micro-LP Nb surfaces are not fully understood.

## 3. Materials and Methods

### 3.1. Materials and Laser Polishing Treatments

Fine-grained niobium sheets measuring approx. 21×21 mm^2^ and 3 mm thickness with 85.25 to 86.8 grains per mm^2^ were laser polished. Test fields, each of 5×5 mm^2^, were treated with different polishing parameters. The samples were produced by using sheets from the series production of TESLA resonators for the European XFEL by high-pressure water jet cutting at DESY (Hamburg, Germany). The untreated surfaces had an average roughness of $R_{a}=\left(0.97-1.43\right)\;µm$ and were laser polished without prior mechanical or chemical polishing [14].

LP experiments were carried out using a combined macro- and micro-LP process with the equipment available at the Fraunhofer Institute for Laser Technology ILT (Aachen, Germany) [22]. The macro-LP was performed using a laser with a wavelength of 1075 nm (redPOWER, SPI Laser Ltd., Southampton, UK). The pulsed laser used for micro polishing was a Q-switched disk laser (TruMicro7050, Trumpf, Ditzingen, Germany) with a wavelength of 1030 nm. Both lasers feature a circular top-hat intensity profile.

Surface processing was realized by means of a galvanometric laser scanner (hurrySCAN30, ScanLab GmbH, Puchheim, Germany) in combination with a 5-axis CNC system. The setup includes a sealed process chamber, allowing LP under an argon atmosphere with a residual oxygen concentration of below 500 ppm. More details of the LP setups are given, e.g., in [23,24,25,26].

The process parameters, laser power ($P_{L}$), feed rate ($v_{s}$), number of polishing scans (*n*), track offset ($d_{y}$), and spot diameter ($d_{L}$), were systematically varied for both the macro- and micro-LP steps. In the case of micro-LP, the influence of the pulse duration was also investigated.

The results of the LP process were evaluated by analyzing the surface roughness using white-light interferometry. The optimized process parameters for macro-LP were $P_{L}=240$ W, n=2, $d_{y}=50\;$µm, $v_{s}=25$ mm/s, $d_{L}=250\;µm$. Based on surfaces pre-polished with these parameters, the process parameters for micro-LP were further optimized. The optimal parameters for micro-LP were determined as $P_{L}=80$ W, $d_{y}=30\;$µm, $v_{s}=1000$ mm/s, $d_{L}=280\;µm$ [14].

**Figure 1:**
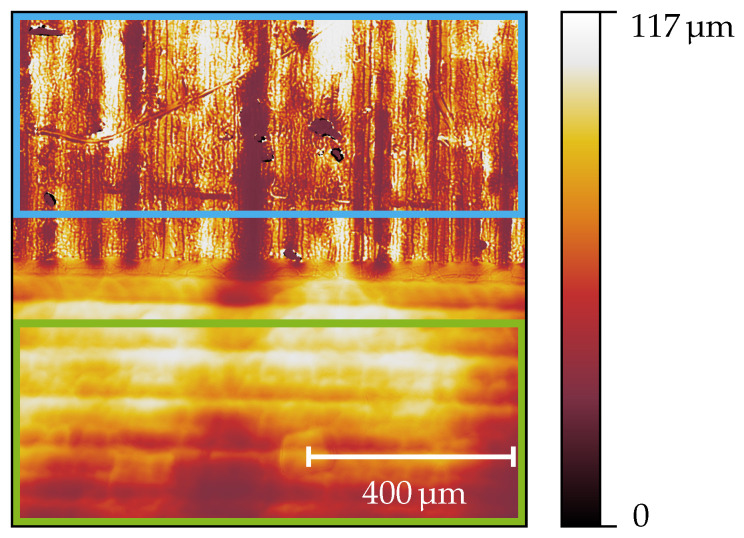
The optical profilometry (OP) image illustrates the smoothing effect achieved by LP. The untreated surface (top, blue-framed) exhibits a root-mean-square areal roughness of $S_{q,\;\mathrm{initial}}=4.59\;$µm. In contrast, the surface processed by the combined macro- and micro-LP (bottom, green-framed) shows a significantly reduced areal roughness of $S_{q,\;\mathrm{LP}}=0.93\;$µm.

Figure 1 shows topographical images acquired by optical profilometry (OP) of the unpolished (NP) area (top) and an LP-treated (LP) area processed using the optimized parameters (bottom). The untreated surface has grooves and holes. The combination of macro- and micro-LP resulted in a significant reduction of the root-mean-square roughness from $S_{q,\;\mathrm{initial}}=4.59\;$µm (blue rectangle) to $S_{q,\;\mathrm{LP}}=0.93\;$µm (green rectangle). LP creates structures through track offset ($d_{y}$) and ripples, which are artifacts of the melt pool. Jumps also occur at grain boundaries. A detailed description of the measurement procedures and a comprehensive analysis of the LP-treated surfaces by scanning electron microscopy (SEM), energy-dispersive X-ray spectroscopy (EDX), and X-ray diffraction (XRD) can be found in [14]. Even under optimized LP conditions, carbon particles, cracking and stress were found on polished surfaces.

### 3.2. Field Emission Methods

**Figure A2:**
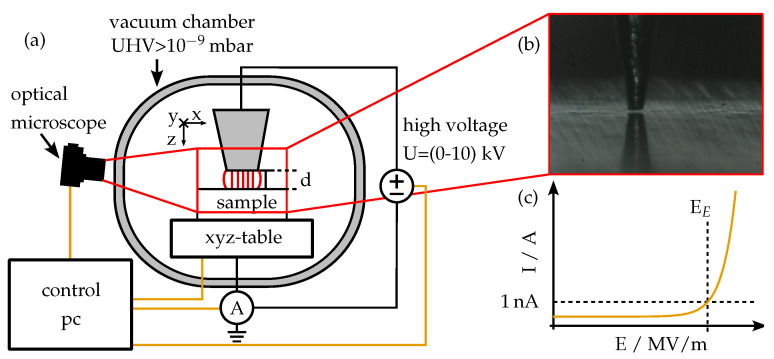
(**a**) Schematic layout of the field emission scanning microscope (FESM). A high voltage is applied between the tungsten anode and the sample under UHV conditions, while the emission current is recorded as a function of the electric field strength. (**b**) Optical micrograph of the measurement anode. (**c**) Typical emission curve following Fowler–Nordheim behavior.

Field emission was examined over a large area (cf. Figure A2) using a field emission scanning microscope (FESM). A high voltage of up to 10 kV is applied between a tungsten anode and the sample in an ultra-high vacuum (UHV, $P<10^{-9}$ mbar). The resulting emission current is recorded as a function of the applied voltage using a high-resolution current measuring device [27]. The distance between the planar anode (diameter 66 µm) and the sample is typically d=(10−15) µm as measured with a long range optical microscope, which results in an almost homogeneous electric field in the measurement range with β≈1 [28]. The sample was positioned using a motorized XYZ table, which enabled precise adjustment and tilt correction relative to the anode. Tilting of the sample or asymmetrically prepared tips can impair field homogeneity and lead to systematic uncertainties [29]. An optical microscope allowed precise adjustment of the anode position relative to the investigated surface (Figure A2b). It should be noted that the measured field emission properties depend on the measurement configuration (e.g., the anode geometry, vacuum conditions, the distance between the anode and the sample, and the criterion for the definition of the onset field), so a direct quantitative comparison between different FESM systems and measurements is not possible.

The measurement methodology of the FESM is based on static electric (DC) fields rather than radio-frequency (RF) fields as present in cavities. Measurements performed under DC conditions nevertheless allow conclusions to be drawn about the parasitic field-emission behavior of surfaces under RF conditions. Reference [30] provides indications that under RF even higher field strengths are required to initiate field emission to a similar extent as under DC. The onset field strengths determined under DC fields can therefore be regarded as a conservative estimate. Key aspects of the fundamental differences between direct current and high-frequency field emission include the influence of time-dependent electromagnetic fields [31], thermal effects [32] and adsorption/desorption processes at the surface during the field emission testing [1]. The temperature dependence of field emission is also important when interpreting results in the context of SRF, since direct-current measurements are performed at room temperature, whereas field emission in the SRF-cavity occurs at operating temperatures of around 2 K [1]. These relationships are discussed in more detail in the Appendix A.2.

Furthermore, DC measurements enable the identification of individual field emitters as well as the determination of their spatial distribution. In addition to surface defects, this may also include contaminants and particles on the surface.

**Figure A3:**
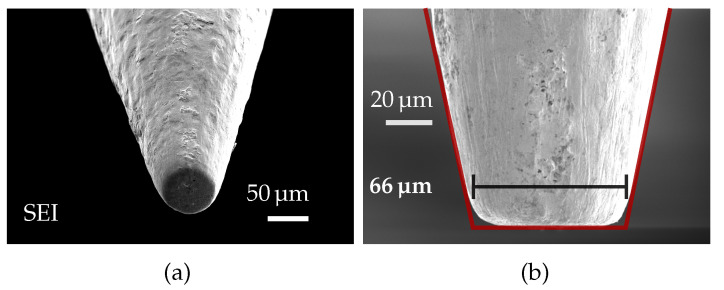
SEM image of the prepared, approximately truncated-cone-shaped tungsten measurement anode with a tip diameter of 66 µm. (**a**) The oblique top view illustrates the geometry and symmetry of the tungsten tips. The side view (**b**) illustrates how the actual tip deviates from the truncated cone shape around the edges.

Figure A3 shows SEM images of the tungsten anode with a diameter of 66 µm prepared for these measurements. The tips were manufactured from a tungsten rod using mechanical and electrochemical process steps. Chemical contaminants were removed by baking the tips under high vacuum conditions for two hours and subsequent activation. To do this, a constant emission current of several hundred nA is applied between the sample and the tip for a period of several minutes. Field emitters on the tip are melted or vaporized by such a activation and deactivation.

For area measurements of the field emission properties, the high voltage is ramped up starting at 0 kV at each measuring position with a linear ramp of 25 V/s, while the emission current $I_{E}$ is continuously measured. The voltage can be converted into the electric field strength E. Plotting the measured emission current $I_{E}$ against the field strength *E* yields an $I_{E}-E$ characteristic curve. This enables the field emission field strength to be determined. In this work the field emission field strengths $E_{E}$ is defined as the field strength at which an emission current of $I_{E}=1\;\mathrm{nA}$ is reached (as shown in Figure A2c). This threshold value is determined purely empirically. It separates the emission current from the measurement noise and background level. The measurement curves for the voltage ramp-up (UP → U) and ramp-down (DOWN → D) are evaluated separately in order to take activation effects into account. For the up- and down-measurements, the field emission field strengths $E_{E,U}$, $E_{E,D}$ are determined. The course of a typical emission curve according to the Fowler–Nordheim theory is shown schematically in Figure A2c. For an entire mapping of a sample, a certain number of measurement points with a suitable spacing in between are recorded.

In area measurements, neighboring measurement points partially overlap, which means that newly formed (i.e., activated) emitters can influence the emission characteristics of subsequent measurements. The measured minimum value of the applied field strength $E_{E}$ may therefore be lower than that of the uninfluenced initial surface. To minimize such effects, it is advisable to limit the applied electric field strength *E* to an upper limit field strength $E_{G}$. However, this method cannot be used to measure the maximum field strength to which a surface can be exposed without field emission occurring. Therefore, we have performed measurements with and without field limitation here.

## 4. Results and Discussion

The field emission properties of untreated and LP surfaces will be discussed in this section. On untreated and LP-treated surfaces, a two-dimensional field emission measurement is performed with 16×16 data points at a lateral distance of approximately 32 µm. For these measurements, we limit the maximum field strength to 400 MV/m. At higher field strengths, breakdowns occur more frequently, leading to irreversible modifications of the surface. This modification results in a reduction of $E_{E}$ and thus distorts the measurements. The LP-treated surfaces in particular exhibit very high activation field strengths, which means that the majority of the emission curves from these measurements cannot be described using the model from Fowler–Nordheim as described in Section 2. The data is therefore evaluated using a classification based on the shape of the measured emission curves. Another measurement on a LP-treated surface with 27×28 measurements (stepsize ≈20 µm) but without field limitation to determine the maximum operating field strength and to verify the relationship between surface structures and emitters is discussed separately.

### 4.1. Clustering of FESM Data

The $I_{E}-E$ curves are classified based on the operating field strengths of the upward measurement $E_{E,U}$ (UP) and the downward measurement $E_{E,D}$ (DOWN). Previous studies have shown that different emitters exhibit characteristic $I_{E}-E$ curves [33], from which the clustering approach in this work has been derived. The classification of the measurement data is performed using Python 3.10.5 scripts developed for this purpose.

**Figure 2:**
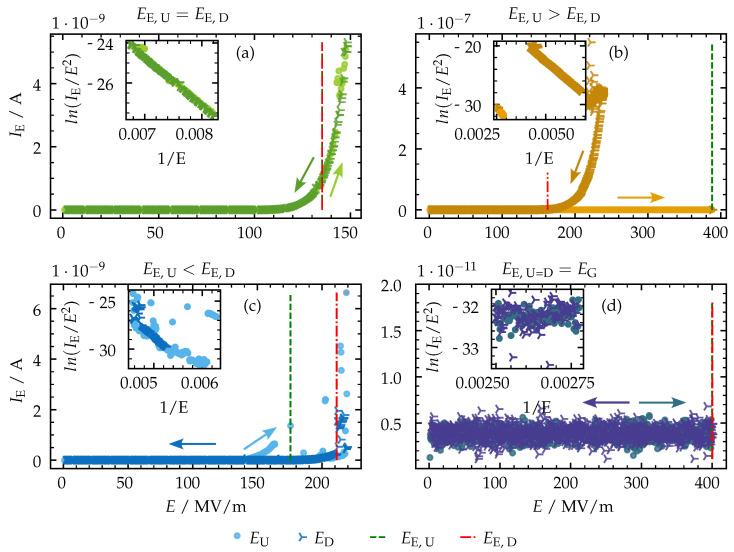
Exemplary $I_{E}-E$ characteristic curves representing the four characteristic emission types. The lighter circular data points correspond to the upward measurements, while the darker triangular markers correspond to the downward measurements. The arrows indicate the increase and decrease of the applied field strength. $E_{E,U}$ is marked in green and $E_{E,D}$ in red. For Fowler–Nordheim-compliant field emission (**a**) $E_{E,U}=E_{E,D}$; for explosive field emission (**b**) $E_{E,U}>E_{E,D}$ holds; for emitter removal (**c**) $E_{E,U}<E_{E,D}$; and for field-limited inactivity (**d**) $E_{E,U}=E_{E,D}=E_{G}$.

Figure 2 shows four measured $I_{E}-E$ characteristics recorded at different positions on the untreated sample. These curves are representative examples for each of the four identified classes. The data are also converted to Fowler–Northeim coordinates (see insets in Figure 2a–d). If the emission current follows Fowler–Nordheim theory with reversible behavior (Figure 2a), i.e., $E_{E,U}=E_{E,D}$; the effective emission area *S* and the field enhancement factor β can be determined from the linear region of the Fowler–Nordheim plot using linear regression. The analysis is limited due to the abrupt change in the emission current in the case of $E_{E,U}>E_{E,D}$ (see Figure 2b) and $E_{E,U}<E_{E,D}$ (see Figure 2c). For some of the electrical measurements, the adaptability of the FN-theory is obviously not feasible due to irreversible activation, deactivation, or explosive electron emission.

If the measurement curves differ in up and down direction distinct physical mechanisms can be identified:

- Explosive field emission ($E_{E,U}\gg E_{E,D}$): No emission current is initially observed despite increasing field strength. Only upon reaching a critical field strength does abrupt activation occur, accompanied by local surface melting and the formation of new emitters (cf. Figure 2b). FN-theory may only be used on the back scan.
- Fowler–Nordheim-compliant field emission ($E_{E,U}=E_{E,D}$): The emission current in up- and down-scans can be described by Equation (2) (cf. Figure 2a).
- Emitter removal ($E_{E,U}<E_{E,D}$): During the upward measurement, an increased emission current ($1\;\mathrm{nA}<I_{E}<$ current limit) is briefly detected before decreasing again. Further increase of the field is necessary to re-achieve emission. This indicates modification or removal of active emitters due to current-induced damage (cf. Figure 2c).

**Figure 3:**
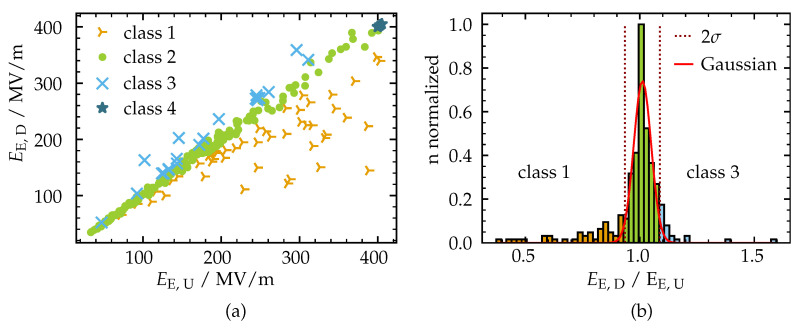
Classification of the classes based on the distribution of the ratio $E_{E,D}/E_{E,U}$ measured on the unpolished (NP) sample. (**a**) Plot of $E_{E,U}$ against $E_{E,D}$ with color-coded class assignment. (**b**) Distribution of the ratio $E_{E,D}/E_{E,U}$, fit with Gaussian of half width 2σ=0.075 including the interval used to determine the class boundaries.

From these considerations, four classes are defined. Class 1 corresponds to explosive field emission ($E_{E,U}>E_{E,D}$), class 2 to reversible Fowler–Nordheim-compliant emission ($E_{E,U}=E_{E,D}$), class 3 to emitter removal ($E_{E,U}<E_{E,D}$), and class 4 to field-limited inactivity ($E_{E,U}=E_{E,D}=E_{G}$, Figure 3d).

**Figure 4:**
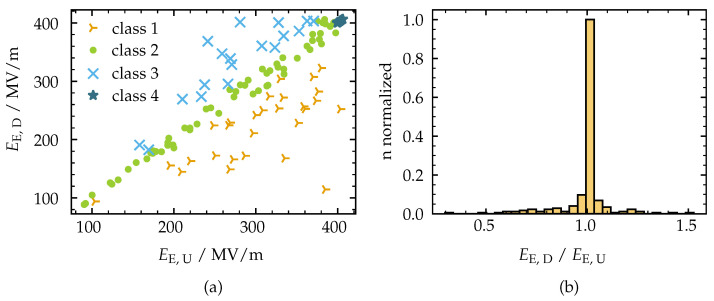
(**a**) Plot of $E_{E,U}$ versus $E_{E,D}$, with color-coded class assignments for the LP-treated surface. A pronounced accumulation of class 4 data points is evident, indicating that no electron field emission was observed, even at the maximum field strength of 400 MV/m. (**b**) Histogram of the $E_{E,D}$/$E_{E,U}$ ratios derived from (**a**).

These four classes provide a systematic framework for comparing the emission behavior across different surface conditions. In particular, the ratio $E_{E,D}/E_{E,U}$ is used for automated classification. According to the Fowler–Nordheim theory, plotting $E_{E,U}$ against $E_{E,D}$ yields a linear relationship with a slope of one. Deviations from this behavior indicate activation processes, new emitters or emitter removal (see Figure 3a). The corresponding distribution can be approximated by a Gaussian distribution as shown in Figure 3b. Measurements within 2σ of the expected value $E_{E,D}/E_{E,U}=1$ are assigned to class 2, unless $E_{E,U}=E_{E,D}=E_{G}$ applies (class 4). Class 1 contains all data with $E_{E,D}/E_{E,U}<1-2\sigma$, class 3 all data with $E_{E,D}/E_{E,U}>1+2\sigma$. For an untreated niobium surface, a Gaussian with 2σ=0.075 was determined. The same four classes are also observed for the LP-treated surface, as demonstrated in Figure 4a for companion. Unlike the untreated surface, the LP surface shows a significantly larger number of measurement points in class 4 (110 compared to 8 for the untreated sample), which substantially distorts the distribution (cf. Figure 4b). For this reason, the classification boundaries based on 2σ=0.075 determined from the untreated surface are maintained. Table 1 shows that class assignment and operating field strength do not necessarily correlate; the assignment is determined solely by the shape of the $I_{E}-E$ curves.

The largest spread in field strength occurs in class 2, as different emitters of varying morphology may emit according to the Fowler–Nordheim theory at different local field strengths. The lowest maximum field strengths are observed in class 3, consistent with the expectation that particle-induced emission is more easily suppressed, as also shown in previous studies [14,34]. Slightly higher values than $E_{G}$ can be attributed to system response inertia. The lower number of data points in class 1 for the LP sample compared to the untreated sample is attributable to field limitation. Although higher electric field strengths generally lead to an increased occurrence of electrical breakdowns (larger number of emitters in class 1), the field limitation in the LP sample prevents field emission from occurring in the first place (resulting in a higher number of points in class 4).

### 4.2. Field Emission Properties of Unpolished and Laser-Polished Surfaces

**Figure 5:**
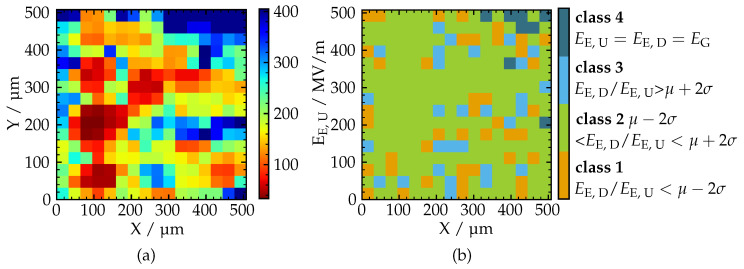
The measurement with 16×16 measuring points shows the emission behavior of an NP-surface. (**a**) Spatial distribution of the onset field strength $E_{E,U}$ with a field limitation of $E_{G}=400$ MV/m. (**b**) Spatial distribution of the four field emission classes on the same measurement surface. The different colors represent the classes 1–4.

**Figure 6:**
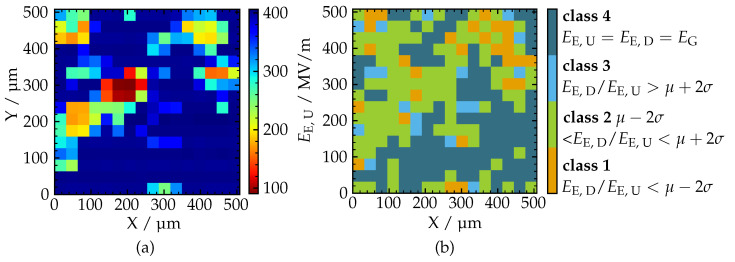
The measurement with 16×16 measuring points shows the emission behavior of a LP-treated surface. (**a**) Spatial distribution of the operating field strength $E_{E,U}$ with a field limitation of $E_{G}=400$ MV/m. (**b**) Spatial distribution of the four identified emission curves based on the ratio $E_{E,D}$/$E_{E,U}$.

**Figure 7:**
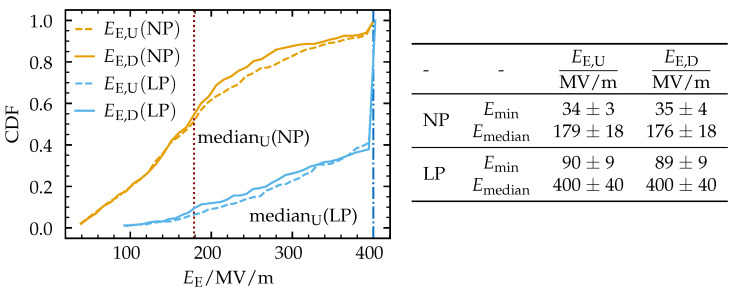
Cumulative density functions of the operating field strengths with field limitation $E_{G}=400\;$ MV/m for untreated (NP) and LP-treated (LP) surfaces. The table shows the minimum and median operating field strengths of the respective distributions.

Figure 5 shows the areal field emission mapping of the NP-niobium surface, whereas Figure 6 presents the corresponding mapping for the LP-treated surface. The statistical characteristics of the operating field strengths are provided in Table 2. The minimum values, medians and cumulative density functions (CDF) are summarized in Figure 7, separately for between UP and DOWN measurements.

Figure 5a reveals spatial structures in the operating field strength distribution, originating from the micro-structure of the pristine sheet material (cf. Figure 1a). Due to the finite anode size (66 µm), these structures appear slightly enlarged. Figure 5b shows the spatial distribution of the emission classes. The distribution of class 3 points is random, consistent with an expected random distribution of contaminant particles, as confirmed by the investigation of several untreated samples. The local accumulation of class 4 points in the upper right region is likely associated with a shallow depression in the surface topography.

The mapping of the activation field strength on the untreated surface shows a heterogeneous distribution of field emission sites. Localized regions with reduced operating field strength are observed predominantly near grain boundaries and surface asperities like grooves and ripples. These sites are characterized by higher field enhancement factors, which lower the threshold for electron emission under high electric field loading.

In contrast, the mapping of the operating field strength on the LP-treated surface exhibits characteristic bands of reduced field strength aligned along diagonal, line-like surface features. These regions coincide with crack structures formed during LP [14]. The reduced activation field strength along these cracks indicates that they act as preferential emission sites due to locally enhanced electric fields.

More than half of the measurement points on the LP surface fall into class 4, meaning that either no field emission is detected or emission occurs only close to the global field limit of $E_{G}=400$ MV/m. This behavior results from the reduction of surface roughness introduced by LP, which decreases local curvature and therefore reduces the effective field enhancement factor. The suppression of small-scale asperities increases the activation field threshold and allows stable operation at higher electric field levels. This trend is consistent with literature reports on LP-treated niobium surfaces, where increased breakdown onset fields are attributed to the smoothing of micro-topographic features [15].

However, unlike the samples investigated in [15], the macro polishing procedure applied here results in the formation of thermally induced cracks. These cracks locally reverse the smoothing effect by reintroducing sharp geometric features with small radii of curvature. As a consequence, emission reappears at significantly lower global field strengths along the crack network. This explains the accumulation of class 1 and class 3 emission sites within the cracked regions. The correlation becomes evident from the overlay of the measured field emission map (Figure 6) and a pre-measurement SEM image of the investigated area (see Figure A6).

The measured crack widths in the range of (60–100) µm are comparable to the lateral resolution limit imposed by the truncated-cone anode geometry (66 µm tip diameter) used in the FESM system. Therefore, the observed emission structures represent a convolution of the actual surface topography and the spatial averaging intrinsic to the measurement setup. Nevertheless, the correlation between crack locations and reduced activation field is clearly resolved in the mapping data. Further research using sharper anodes remains a subject of future work.

Field emission is not exclusively associated with cracks. SEM inspection after mapping reveals embedded carbon-containing inclusions introduced during the LP process. These particles act as additional emission sites and follow Fowler–Nordheim characteristics. Their presence accounts for isolated class 3 emission sites, that occur outside the crack network. A comparison of the CDF in Figure 7 shows that LP leads to a significant increase in the operating field strengths: the minimum measured value approximately doubles to triples, and the median also increases significantly. A comparison of the UP and DOWN measurements shows that the surface was only slightly modified by the field limitation. The largest deviations occur in the field strength range from approximately 190 MV/m to 340 MV/m, with this effect being more pronounced on the untreated surface than on the laser-polished surface.

To further characterize the emission sites of the laser-polished Nb surface, the measured current-field characteristics were analyzed in Fowler–Nordheim (FN) coordinates according to Equation (4). In the following, only the field-emission characteristics of the laser-polished surface are analyzed. The corresponding spatial distribution of the onset field strengths is shown in Figure 6. The field enhancement factor β and the effective emission area *S* were subsequently determined from the slope and intercept of the linear regression using Equations (7) and (8), respectively. Throughout the analysis, the work function was assumed to be Φ=4.3 eV, the reported work function of polycrystalline niobium [35]. This assumption represents an approximation, since surface contamination, native oxides (Nb_2_O_5_, reported work function 5.2 eV) and, in particular, carbon-containing particles may exhibit different work functions. Moreover, the local crystal structure at the surface as well as the presence of surface oxides may additionally affect the work function of the niob surface [36,37,38]. Consequently, the extracted values of β and *S* should be regarded as effective parameters. According to Equation (7), the field enhancement factor scales with the work function as $\beta \propto \Phi^{3/2}$, whereas the effective emission area follows $S\propto \Phi^{-2}$. Therefore, uncertainties in the assumed work function primarily affect the absolute values of these parameters, while the relative differences between emitters remain meaningful.

A Fowler–Nordheim analysis is only applicable to emitters exhibiting stable emission behavior. Emitters showing pronounced activation, irreversible modification or electrical breakdown during the voltage sweep were therefore excluded, since their current-field characteristics cannot be adequately described by Fowler–Nordheim theory. To minimize the influence of the background current, only data points with emission currents exceeding 0.5 nA were considered for the linear regression. Furthermore, only fits with a negative slope and a coefficient of determination greater than $R^{2}=0.5$ were accepted. The FN analysis was performed independently for the up- and down-sweep measurements, allowing both the initial emitter population and electrically activated emitters to be characterized.

**Figure 8:**
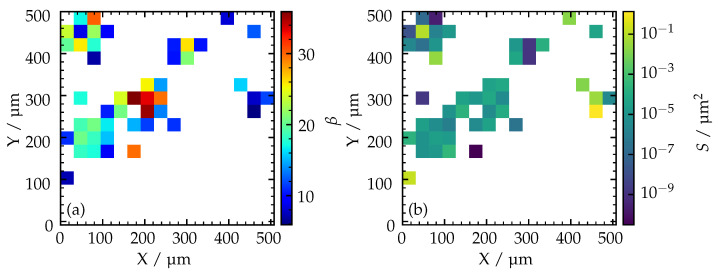
The spatial distribution of the field enhancement factor β (**a**) and the effective emission area *S* (**b**), obtained from Fowler–Nordheim analysis of up-sweep measurements taken from the laser polishing surface (see Figure 6). Only emitters exhibiting stable emission behavior that can be adequately described by Fowler–Nordheim theory are included in the analysis.

**Figure 9:**
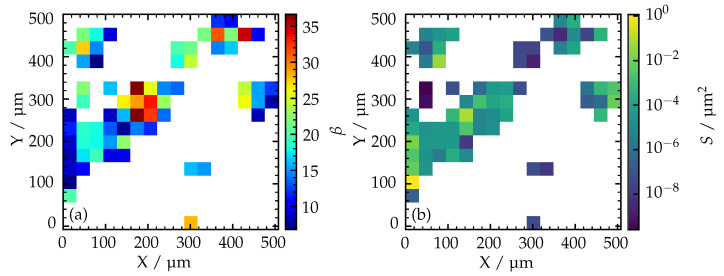
The spatial distribution of the field enhancement factor β (**a**) and the effective emission area *S* (**b**), obtained from Fowler–Nordheim analysis of up-sweep measurements taken from the laser polishing surface after emitter activation (see Figure 6). The analysis includes only emitters whose current-field characteristics are consistent with Fowler–Nordheim theory. Comparison with Figure 8 illustrates the changes in the emitter population following electrical activation.

The spatial distributions of the extracted field enhancement factors and effective emission areas obtained from the up-sweep measurements are shown in Figure 8, while the corresponding distributions for the down-sweep measurements are presented in Figure 9. During the up-sweep, 47 of the 256 measured emission curves fulfilled the FN fitting criteria. Following electrical activation, this number increased to 69 during the down-sweep, indicating that additional emitters became active only after exposure to higher electric fields.

For the up-sweep measurements, the extracted field enhancement factors range from approximately β=6 to 36, while the effective emission areas are in the range from 1.4 µm^2^ to $3\times 10^{-11}$ µm^2^. After electrical activation, the field enhancement factors span a comparable range of approximately β=8 to 37, whereas the effective emission areas increase to values in the range from 1.1 µm^2^ to $2\times 10^{-10}$ µm^2^. Although the absolute values depend on the assumed work function, the observed spatial distributions and relative differences between emitters are unaffected.

The analyzed emitters are not randomly distributed over the sample surface but are concentrated in regions where SEM investigations revealed remelted niobium defects and carbon-containing surface residues. The highest field enhancement factors (β>30) are preferentially found at locations where carbon-containing particles were previously identified. This observation is consistent with the pronounced local field enhancement expected from sharp particulate contaminants. In contrast, emitters associated with remelted niobium defects generally exhibit lower field enhancement factors, suggesting a comparatively smoother emitter geometry and therefore weaker local field concentration.

The effective emission area does not exhibit such a pronounced spatial localization. This behavior is expected, since *S* represents an effective parameter of the Fowler–Nordheim model rather than the true geometrical emitter area. Its absolute value is influenced not only by the emitting area itself but also by the assumed work function and the simplifying assumptions of the Fowler–Nordheim model. Consequently, the effective emission area should primarily be regarded as a comparative parameter describing the apparent extent of the active emission region rather than the physical size of an individual emitter.

Comparison of the up- and down-sweep analyses further demonstrates that electrical conditioning modifies the active emitter population. Besides the increased number of analyzable emitters, the occurrence of higher field enhancement factors after the application of elevated electric fields suggests that previously inactive emission sites become activated or that existing emitters undergo morphological modifications in agreement with previous investigations. When combined with the SEM observations and the defect classification presented in the previous section, the Fowler–Nordheim analysis indicates that different defect classes exhibit distinct emission characteristics. In particular, carbon-containing particles are associated with the highest field enhancement factors, whereas remelted niobium defects typically exhibit lower values. These results suggest that the dominant field-emission behavior is governed primarily by the local defect morphology, with sharp particulate contaminants producing significantly stronger local electric field enhancement than the comparatively smooth remelted niobium defects.

**Figure 10:**
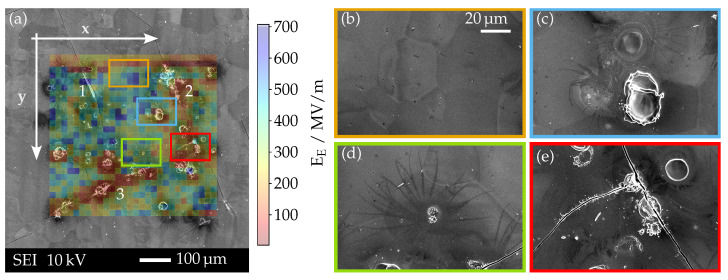
(**a**) Spatial correlation between the measured activation field strength and post-measurement SEM images of the same region. Numbers 1–3 indicate three cracks that already existed prior to the field emission measurement as a result of the LP. Panels (**b**–**e**) correspond to the colored regions highlighted in (**a**). (**b**) Region without detectable field emission-induced modification. (**c**) Region showing surface modification caused by a breakdown event. (**d**,**e**) regions where field emission originates from defect structures such as cracks or embedded particles.

A further areal FESM measurement was carried out without field limitation. The measurement comprises 27×28 measuring points, whereby only the operating field strengths were stored; an analysis of the complete $I_{E}-E$ curves was therefore not possible. Figure 10a shows the superposition of a SEM image of the LP-treated surface after the field emission measurement with the mapping of the operating field strengths. The partial images Figure 10b–e show areas with typical surface structures that have been altered by field emission. Table 2 contains mean values, standard deviations and minimum and maximum operating field strengths for the four excerpts.

**Table 2:** Statistical values $E_{\mathrm{mean}}$, $E_{\mathrm{std}}$, $E_{\max}$ and $E_{\min}$ for the four sections b–e in Figure 10. The nature of the surface in the respective areas obviously influences the measured field emission parameters.

Areas	$E_{E,U,\;\mathrm{mean}}$/MV/m	$E_{E,U,\;\mathrm{std}}$/MV/m	$E_{E,U,\;\max}$/MV/m	$E_{E,U,\;\min}$/MV/m
(b)	347	144	613	76
(c)	292	128	554	7
(d)	348	94	573	134
(e)	202	82	386	95

Overall, however, there is a clear correlation between the surface structure and the operating field strength. Low values are typically due to defects such as cracks or areas modified by stress breakthroughs. Maximum operating field strengths of up to (674 ± 67) MV/m were achieved at specific points, with an average of (294 ± 29) MV/m. In area (b), the surface shows only minor modifications despite an applied field strengths above 600 MV/m, while in (c) significant structural changes occur. The minimum operating value of 7 MV/m in (c) is due to these modifications. Area (d) shows only moderate changes, which allows for higher mean operating field strengths. In (e), it can be seen that field emission occurs preferentially along the cracks, making it clear that these are to be regarded as the main cause of the reduced operating field strength of the laser-polished sample.

In general the surface roughness of the niobium samples was reduced by a two-step laser polishing process consisting of continuous-wave (CW) laser polishing followed by nanosecond laser polishing [14]. Both processes involve localized energy deposition into the near-surface region, resulting in the formation of a melt pool and a heat-affected zone (HAZ) that may lead to the smoothing after resolidification. Owing to the significantly shorter laser–material interaction time, nanosecond laser polishing introduces a lower overall heat input than CW laser polishing, thereby reducing the depth of the HAZ [39,40]. Nevertheless, both processes are characterized by rapid heating and cooling cycles that generate steep temperature gradients between the molten surface layer and the underlying bulk material. The resulting constrained thermal expansion during heating and contraction during solidification induce thermomechanical stresses. If these stresses exceed the local yield strength or fracture resistance, residual stresses may accumulate and promote crack initiation [40,41,42].

In the combined CW and nanosecond laser polishing process, the final stress state is governed by the interaction of melt pool dynamics, cooling rate, and repeated thermal cycling. While CW laser polishing produces a comparatively large melt pool and deeper thermal penetration, nanosecond laser polishing locally remelts the surface with rapid solidification. The superposition of both thermal cycles can therefore lead to stress accumulation, particularly in regions subjected to repeated remelting, which may explain the crack formation observed in the present study [40,42].

Several approaches can be employed to mitigate laser-induced residual stresses. Preheating the substrate reduces the temperature difference between the processed surface and the bulk material, thereby lowering thermal gradients and thermally induced strains. In addition, optimization of the laser processing parameters, including laser power, pulse energy, scanning speed, and track overlap, can reduce the overall heat input and limit the extent of the heat-affected zone [42]. Finally, post-process stress-relief heat treatments, such as thermal annealing, may further reduce residual stresses and thereby decrease the susceptibility to crack formation [41,43].

Field emission measurements performed on niobium surfaces finished by buffered chemical polishing (BCP) followed by nanosecond laser polishing exhibited onset fields of up to 500 MV/m without the occurrence of laser-induced cracking (Figure A5). This observation suggests that replacing the initial CW laser polishing step with mechanical or chemical polishing techniques could substantially reduce the cumulative thermal load introduced during surface finishing. Consequently, the generation of residual stresses and the associated crack formation may be suppressed while preserving the high field emission performance achieved by the subsequent nanosecond laser polishing process.

## 5. Summary

**Figure A4:**
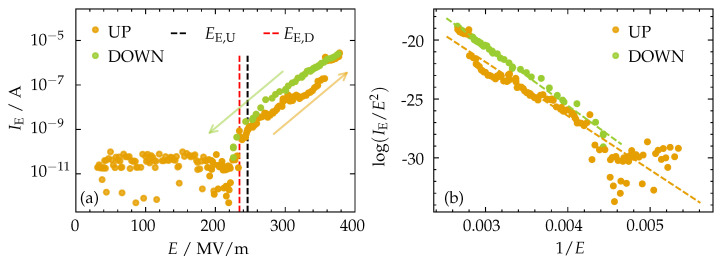
The field emission properties of buffered chemical polishing niobium surfaces. The sample was prepared at DESY according to defined standards. (**a**) shows the evolution of the $I_{E}-E$ curve. (**b**) illustrates the evolution of the data after it has been transformed into the Fowler-Nordheim coordinate system.

**Figure A5:**
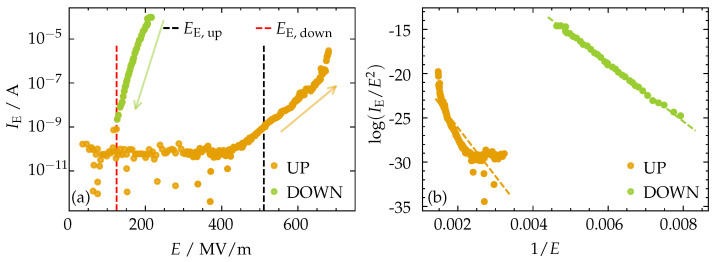
The field emission properties of a laser-polished surface that has been smoothed by buffered chemical polishing are shown here. This polishing is performed at DESY in accordance with established standards. (**a**) shows the evolution of the $I_{E}-E$ curve. (**b**) illustrates the evolution of the data after it has been transformed into the Fowler-Nordheim coordinate system.

**Figure A6:**
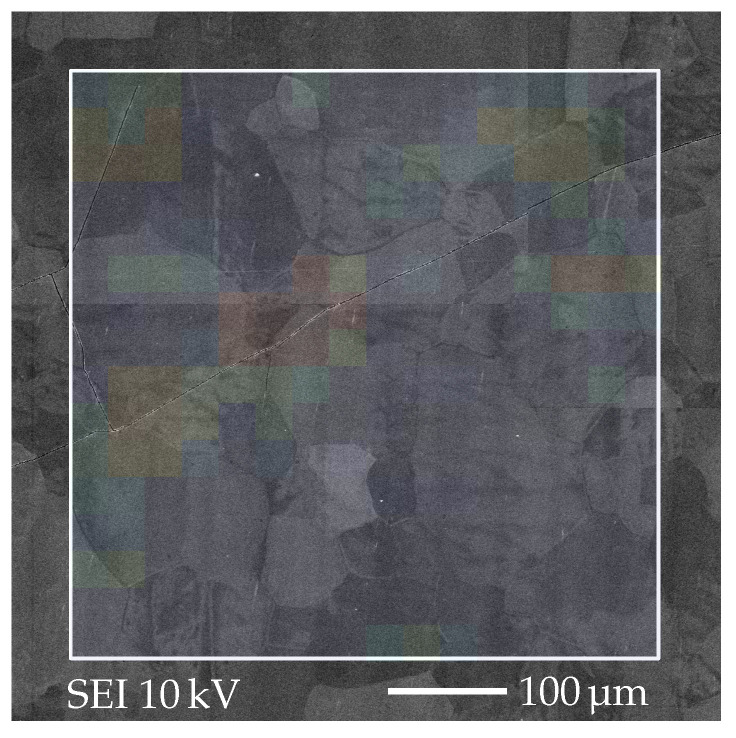
Overlay of the field emission map (Figure 6) and a pre-measurement SEM image of the investigated area.

The field emission properties of unpolished niobium with a total of 256 measurement points and LP-treated surfaces with 1012 measurement points were systematically investigated through combined field emission OP and SEM experiments. Macro- and micro-LP of technically rough niobium sheets leads to a significant increase in the operating field strengths for electron field emission compared to untreated sheets. This is mainly due to the effective reduction in roughness and the removal of sharp surface features, as shown by optical profilometry and electron scanning microscopy. In measurements with a field strength limited to 400 MV/m, a minimum operating field strength of (90±9) MV/m was determined, which can be clearly attributed to defects remaining after LP. On the LP-treated surfaces, operating field strengths of over 700 MV/m were measured at defect-free locations without field limitation, which represents a significant increase compared to typical values for chemically polished niobium surfaces. Systematic direct-current scanning measurements on electrochemically polished (EP) fine grained niobium samples yielded operating field strengths in the range of approximately 60 MV/m, employing the same experimental setup as in the present study [10,44]. Earlier measurements on chemically polished single crystals yielded local operating field strengths of up to 200 MV/m. In good agreement with those previous studies, exemplary measurements on BCP fine grained niobium samples using the measurement methods described here yielded maximum operating field strengths of up to 250 MV/m (Figure A4) [45]. Furthermore, the field enhancement factors (β) as well as the emission area (*S*) determined from these data are well in agreement with β≈12−13 and $S\approx \left(5-90\right)\times 10^{-5}$ µm^2^. The results of this study thus demonstrate that the field emission properties of a defect-free, laser-polished surface are superior to those of a chemically polished surface.

However, crack formation on LP-treated surfaces is a major problem, not only in terms of parasitic field emission, but also with regard to their use in cavity manufacturing. The work of Porshyn et al. showed that, with the appropriate choice of laser parameters, field emission is already possible at operating field strengths of (650−940) MV/m, while excessively high laser fluence or an excessive number of pulses result in emission-active structures with operating fields in the range of (70−190) MV/m [15]. This reduction is due to the strength of the laser-induced structures, and is consistent with previous results. Thus, laser polishing parameters have to be very carefully optimized in any case.

In summary, LP is a promising approach for improving the field emission resistance of niobium surfaces, but process optimization (laser parameters, scanning strategy) and measures to avoid crack- and particle-related defects are necessary before LP can be widely used in large-scale production of SRF cavities. In order to reduce crack formation, the laser macropolishing has to be further optimized, e.g., by adjustment of the temperature profile, or by using alternative polishing techniques in combination with the laser micro-polishing.

The classification of the measured $I_{E}-E$ characteristics enables the discrimination of different types of field emitters and provides complementary information beyond conventional Fowler–Nordheim analysis. While Fowler–Nordheim analysis is restricted to emitters exhibiting stable emission behavior that can be adequately described by Fowler–Nordheim theory, the proposed classification approach also captures emitter activation, deactivation, and other non-stationary emission processes. At the same time, Fowler–Nordheim analysis remains an essential tool for characterizing stable emitters by providing quantitative parameters such as the field enhancement factor and effective emission area. The combination of both methods therefore enables a more comprehensive characterization of field-emission behavior than either approach alone.

## Figures and Tables

**Table 1:** Statistical properties of the operating field strengths $E_{E,U}$ for the four classes, separately for the unpolished (NP) and laser polished (LP) samples: $E_{\mathrm{mean}}$, standard deviation $E_{\mathrm{std}}$, minimum $E_{\min}$, maximum $E_{\max}$, and percentage of elements *n*. A systematic uncertainty of 10% is assumed for the operating field strength.

-	Class	$E_{E,U}$/MV/m				
		$E_{\mathrm{mean}}$	$E_{\mathrm{sth}}$	$E_{\min}$	$E_{\max}$	n/%
NP	c_1_	263	85	72	402	16
	c_2_	173	94	34	405	69
	c_3_	177	62	47	311	11
	c_4_	401	1	400	403	3
LP	c_1_	303	68	103	403	11
	c_2_	322	96	90	404	39
	c_3_	276	61	158	371	7
	c_4_	402	2	399	406	43

**Figure A7:**
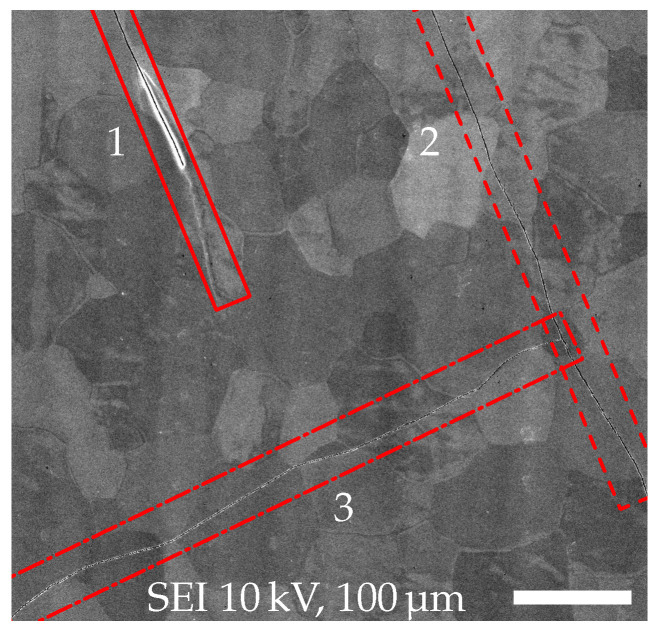
The SEM image shows the condition of the LP-treated surface prior to the surface measurement of the activation field strength (see Figure 10). The grain structure is visible and the three cracks are marked in red.

**Figure A8:**
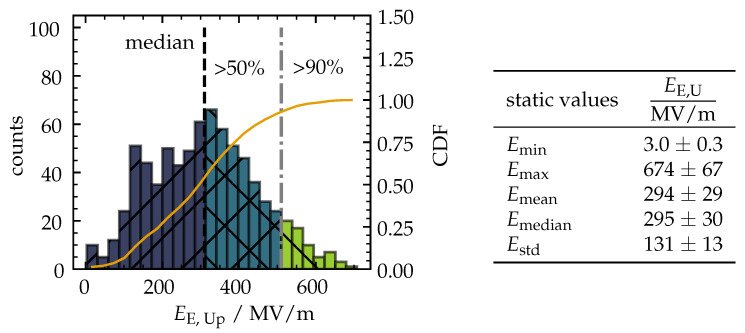
Histogram and cumulative density functions of the onset field strength of the measurement contained in Figure 10.

## Data Availability

The original contributions presented in this study are included in the article. Further inquiries can be directed to the corresponding authors.

## Appendix A

Figure A1 illustrates the quantum-mechanical tunneling process through a finite potential barrier under the influence of an electric field. Figure A2 presents a schematic of the FESM setup and measurement principle of the field emission scanning microscope used to determine the DC turn-on field strength via a high-voltage supply and a tungsten anode. Figure A3 shows SEM images of the prepared tungsten measurement anode employed for the field-emission experiments. The $I_{E}-E$ curve measured on a chemically polished Polycrystalline surface is shown in Figure A4, while the field enhancement factor β determined from the FN plot and the effective emission area *S* are listed in Table A1. Figure A5, on the other hand, shows the $I_{E}-E$ curve measured on a chemically polished sample that was subsequently micro-laser polished. The *S* and β values are listed in Table A2. Figure A6 presents an overlay of the field emission map (Figure 6) and a pre-measurement SEM image of the investigated area, demonstrating the correlation and the robustness of the applied mapping approach. Figure A7 presents an SEM image of an LP-treated surface prior to its characterization with the FESM (cf. Figure 10). The corresponding distribution of the measurement data is given in Figure A8. The differences between DC field emission measurements and field emission mechanisms in RF fields are discussed in Appendix A.2.

### Appendix A.1. Supplementary Information and Measurement Data

**Table A1:** The field enhancement factors β and the effective emission areas *S* were determined from the Fowler–Nordheim linearization of the measured BCP fine-grained sample in Figure A4.

	$\beta_{U}$	$S_{U}$/µm^2^	$\beta_{U}$	$S_{U}$/µm^2^
BCP	12.8	$4.9\times 10^{-5}$	11.7	$8.6\times 10^{-4}$

**Table A2:** The field enhancement factors β and the effective emission areas *S* were determined from the Fowler–Nordheim linearisation of the measured BCP fine-grained sample in Figure A5.

	$\beta_{U}$	$S_{U}$/µm^2^	$\beta_{U}$	$S_{U}$/µm^2^
BCP	10.7	$6.3\times 10^{-8}$	18.0	$1.8\times 10^{-1}$

### Appendix A.2. Relevance and Limitations of DC Field-Emission Measurements for SRF Cavities

Although the FESM operates under static electric (DC) fields, the obtained field-emission characteristics are highly relevant for superconducting radio-frequency (SRF) cavities because the underlying emission mechanism is the same. In both DC and RF operation, electron emission is governed by Fowler–Nordheim (FN) tunneling from localized surface features with enhanced electric fields [16,46,47]. Consequently, parameters such as the effective field enhancement factor β primarily reflect the intrinsic emission characteristics of a surface and are therefore expected to be comparable for both excitation modes. The experimentally determined onset field, however, additionally depends on the measurement configuration, including the field geometry, detection threshold and measurement procedure, and should therefore only be compared qualitatively between different experimental setups [19].

For a sinusoidal RF field,

(A1) $$\begin{array}{c} E\left(t\right)=E_{0}\sin\left(\omega t\right), \end{array}$$

the emitted current is obtained by averaging the Fowler–Nordheim current over one RF cycle. Compared with the DC case, this time averaging mainly modifies the proportionality factor and slightly changes the effective field dependence, while the exponential Fowler–Nordheim parameter remains essentially unchanged. Consequently, similar field enhancement factors are generally obtained from DC and RF measurements, in agreement with experimental observations reported in the literature [48].

Despite the identical emission mechanism, several important differences exist between DC and RF field emission. Unlike in DC measurements, electrons emitted in an RF cavity experience time-dependent electromagnetic fields. Depending on the RF phase at emission, electrons may be accelerated away from the surface or return to it, giving rise to additional processes such as impact heating, secondary electron generation and X-ray production. These effects contribute to cavity performance limitations but are not directly probed by DC field-emission measurements [30,49,50].

Thermal effects constitute another important distinction. Under continuous DC excitation, local heating is primarily determined by the stationary emission current. In contrast, RF-induced heating strongly depends on the pulse duration and duty cycle. Experimental investigations have shown that RF pulses shorter than approximately 2 ms produce only negligible heating of emitting particles, whereas longer pulses or continuous-wave (CW) operation can result in significant local temperature rise, potentially modifying or even eliminating field emitters through melting, restructuring or oxidation [30,50]. These observations are consistent with the experimentally observed dependence of RF conditioning on pulse length.

The surface condition may also evolve differently under RF operation. Local heating, electron bombardment and repeated RF cycling can promote adsorption and desorption processes as well as changes in the surface oxide, thereby modifying the local work function or altering the geometry of individual emission sites. As a result, the emission behavior of a given emitter may evolve during cavity operation. In contrast, DC measurements predominantly characterize the initial emission behavior of a comparatively stable surface state [7,49].

Another important difference is the operating temperature. The FESM measurements presented in this work are performed at room temperature, whereas SRF cavities typically operate at approximately 2 K. Cryogenic temperatures influence surface diffusion, defect mobility and the stability of adsorbates. Nevertheless, the Fowler–Nordheim tunneling process itself depends primarily on the local electric field and the electronic surface potential and is therefore only weakly temperature dependent. Consequently, the intrinsic emission mechanism remains unchanged, although the stability and long-term evolution of individual emitters may differ between room-temperature DC measurements and cryogenic RF operation [1].

Overall, DC field-emission measurements should be regarded as a characterization method for the intrinsic emission properties of surface defects rather than as a direct quantitative predictor of SRF cavity performance. They provide a reproducible basis for comparing different surface treatments and a conservative indication of the intrinsic field-emission behavior under well-defined experimental conditions. The quantitative emission behavior in operating SRF cavities, however, is additionally influenced by RF-specific processes, including the time dependence of the electromagnetic field, electron dynamics, thermal effects and surface evolution [1].
